# Favorable coronary outcomes following IL-6 blockade with tocilizumab in IVIG-resistant kawasaki disease: a case series

**DOI:** 10.3389/fimmu.2026.1729717

**Published:** 2026-01-29

**Authors:** Jing Zheng, Yu Xu, Yan Pu, Jingyue Liu, Zhilang Cao, Yajun Wang

**Affiliations:** 1 Department of Pediatrics, The First People’s Hospital of Yunnan Province, Kunming, China; 2 Department of Pediatrics, The Affiliated Hospital of Kunming University of Science and Technology, Kunming, China; 3 Department of Rheumatology, TengChong People’s Hospital, TengChong, China; 4 Department of Pediatrics, Yunnan Honghe Prefecture Central Hospital, HongHe, China

**Keywords:** coronary artery dilation, IL-6 receptor antagonist, IVIG-resistant, kawasaki disease, tocilizumab

## Abstract

**Objective:**

To evaluate the efficacy and coronary outcomes of tocilizumab (TCZ) in patients with intravenous immunoglobulin (IVIG)-resistant Kawasaki disease (KD), and to place these findings against the conflicting available evidence.

**Methods:**

We retrospectively analyzed four patients with IVIG-resistant KD who received TCZ as rescue therapy. Clinical, laboratory, and echocardiographic data (coronary artery Z-scores) were collected before and after treatment.

**Results:**

All patients received a second dose of IVIG before TCZ administration, however, symptoms did not improve. Following TCZ administration (median 15.5 days from onset), all patients achieved normothermia within 24 hours, accompanied by rapid normalization of inflammatory markers. Notably, no new coronary artery lesions (CALs) were identified after treatment. Additionally, in the two patients with pre-existing CALs, complete resolution of coronary dilation was observed during follow-up. No drug-related adverse events occurred.

**Conclusion:**

In this series, late administration of TCZ during the subacute phase was associated with a rapid anti-inflammatory response and favorable coronary remodeling, including aneurysm regression. This outcome contrasts with previous reports of coronary dilation following earlier intervention. Critically, these observations lead us to propose a “phase-dependent efficacy” hypothesis: the timing of IL-6 blockade relative to disease stage may be a critical determinant of coronary outcomes. This hypothesis underscores the need to consider the disease phase when evaluating IL-6 blockade for refractory KD.

## 1 Introduction

Kawasaki disease (KD) is an immune-mediated small and medium-vessel vasculitis that primarily affects children under 5 years old. Its most severe complication is damage to the coronary arteries (1). Coronary artery lesions (CALs), including coronary-artery dilatation and coronary-artery aneurysms, occur in about 25% of untreated KD and are the primary cause of acquired heart disease in children (1). The administration of IVIG reduces the incidence of coronary artery aneurysms to 4% (2, 3), and the most important determinant of CALs development is the timing of IVIG administration (4). However, 10% to 20% of children are resistant to IVIG, and the incidence of CALs ranges from 19% to 40% (5, 6).

Currently, there is no unified treatment for children with IVIG-resistant KD, which mainly includes the second dose of IVIG, glucocorticoids, monoclonal antibodies, or cytotoxic drugs (1). With ongoing research into the pathogenesis of KD, biological agents are gradually being used to treat the condition. It has been shown that KD is characterized by a cytokine storm, with elevated levels of tumor necrosis factor-α (TNF-α), interleukin-6 (IL-6), interleukin-1β (IL-1β), interleukin-17 (IL-17), and other cytokines during the acute stage (7). IL-6, a pleiotropic cytokine, plays a significant role in various inflammatory and immune-related disorders, including KD. Clinical data indicate that IL-6 levels are significantly higher in the IVIG-resistant KD group than in the IVIG-responsive group, suggesting an association between IL-6 overexpression and IVIG resistance (8, 9). At present, studies on the treatment of KD with biologics primarily focus on anti-TNF-α monoclonal antibodies (e.g., Infliximab) and IL-1 receptor blockers (e.g., Anakinra) (10–15), whereas there are few reports on the efficacy and safety of tocilizumab (TCZ, an IL-6 receptor antagonist) in treating KD.

IL-6 plays a pivotal role not only as an inflammatory mediator but also as a direct driver of vascular endothelial injury through its unique “trans-signaling” pathway. In this process, IL-6 binds to its soluble receptor (sIL-6R), forming a complex that activates vascular endothelial cells that express glycoprotein 130 (gp130). This leads to the upregulation of adhesion molecules (e.g., Vascular Cell Adhesion Molecule-1(VCAM-1), Intercellular Adhesion Molecule-1(ICAM-1), increased vascular permeability, and recruitment of neutrophils and other inflammatory cells into the vessel wall, culminating in coronary arteritis, dilation, and potential aneurysm formation (16). Furthermore, IL-6 disrupts immune homeostasis by downregulating regulatory T cells (Tregs) and promoting the proinflammatory T helper 17 (Th17) cell response, thereby exacerbating an autoimmune-like attack on the vascular wall (17, 18). Consequently, IL-6 serves as a critical molecular link between systemic inflammation and coronary artery damage.

However, the emerging clinical experience with TCZ in KD presents a paradox regarding its coronary safety profile. The seminal report by Nozawa et al. raised an initial concern by documenting new coronary artery aneurysms in two of four patients treated with TCZ early in the disease course (median day 7). More recently, Ling et al. reported a case of progressive coronary dilation in one patient with severe, multidrug-resistant disease. These observations stand in contrast to our present series, which documented favorable coronary outcomes. This inconsistent and conflicting evidence on whether IL-6 blockade mitigates or, in some cases, appears associated with coronary deterioration creates a critical dilemma for clinicians. The timing of TCZ administration relative to the dynamic immunopathological stages of KD may be a key, underappreciated variable that could reconcile these disparate findings.

Therefore, this small case series aims to preliminarily evaluate the clinical utility of TCZ as salvage therapy in children with IVIG-resistant KD, with a focused investigation on its coronary safety profile. Our specific objectives are: 1) To assess the rapidity and consistency of TCZ in controlling systemic inflammation; 2) To meticulously describe the evolution of coronary artery Z-scores before and after treatment, with particular attention to the incidence of new CALs and the fate of pre-existing lesions; and 3)The central and culminating objective is to critically compare our findings with the conflicting reports in the literature. By specifically analyzing the potential link between the timing of TCZ administration and coronary outcomes, we seek to generate clinical evidence for or against the “phase-dependent efficacy” hypothesis. This exploratory analysis aims to provide a novel framework for reconciling disparate data and to inform the design of future stage-stratified studies.

## 2 Patients and methods

### 2.1 Study population

This retrospective study analyzed four patients with IVIG-resistant KD admitted to the Department of Pediatrics at The First People’s Hospital of Yunnan Province between December 2019 and March 2021.

### 2.2 Patient selection

#### 2.2.1 Definition of IVIG resistance

IVIG-resistant cases were defined as those with persistent fever (>36 hours post-IVIG infusion) or recurrence of KD-related fever after initial defervescence (1).

#### 2.2.2 Inclusion criteria

Patients were enrolled if they met the following criteria (1): (1) Diagnosis of either complete or incomplete KD according to the 2017 American Heart Association (AHA) criteria. (2) Met the above study definition of IVIG resistance after the first IVIG course.

(3) Had complete clinical, laboratory, and serial echocardiographic data available from the acute phase through at least a 3-month follow-up.

#### 2.2.3 Exclusion criteria

Patients were excluded if they: (1)had received any immunosuppressive agents (e.g., cyclosporine, cyclophosphamide) or biologic agents (e.g., infliximab) before the administration of TCZ; (2) had a confirmed diagnosis of other rheumatic or autoimmune diseases (e.g., systemic juvenile idiopathic arthritis) that could mimic or confound the diagnosis of KD; (3) presented with clinical or laboratory features suggestive of Macrophage Activation Syndrome (MAS) at the time of TCZ consideration; (4) had evidence of concurrent serious bacterial, viral (including confirmed COVID-19), or fungal infection requiring antimicrobial therapy at the time of TCZ administration; (5) had a known history of congenital heart disease or pre-existing coronary artery anomalies.

#### 2.2.4 Differential diagnosis workup

To ensure diagnostic accuracy, all enrolled patients underwent a standardized evaluation to exclude mimickers. This included:(1) For Systemic Juvenile Idiopathic Arthritis (sJIA): Assessment for typical quotidian fever pattern, evanescent rash, and arthritis, guided by the 2024 EULAR/PReS recommendations (19); (2) For MAS/HLH: Serial monitoring of ferritin, fibrinogen, triglycerides, and platelet counts, as per the 2022 EULAR/ACR classification criteria for HLH/MAS (20); (3) For Infections: Routine bacterial cultures, viral PCR panels (e.g., adenovirus, Epstein-Barr virus), and SARS-CoV-2 testing were performed as clinically indicated.

### 2.3 Study design and data collection

#### 2.3.1 Clinical and laboratory evaluation

Data were collected before and after TCZ administration, including: (1) Fever duration and patterns (pre- and post-treatment); (2) Serial laboratory markers (at admission and post-TCZ); (3) Echocardiographic assessment of CALs.

#### 2.3.2 Coronary artery assessment

Coronary artery damage was assessed using echocardiogram results. The coronary artery Z-score was calculated for each child based on gender, age, weight, and echocardiographic data (1). CAL severity was classified as follows: (1) No dilation: Z-score < 2.0; (2) Coronary dilation: 2.0 ≤ Z-score < 2.5; (3) Small aneurysm: 2.5 ≤ Z-score < 5.0; (4) Medium aneurysm: 5.0 ≤ Z-score < 10.0; (5) Giant aneurysm: Z-score ≥ 10.0.

#### 2.3.3 Therapeutic protocol

Eligible patients received a single dose of TCZ, administered intravenously in accordance with sJIA dosing guidelines (19): 12 mg/kg for body weight <30 kg, 8 mg/kg for body weight ≥30 kg.

#### 2.3.4 Statistical analysis

Descriptive statistics (means, proportions) were used to summarize clinical and laboratory parameters. Temporal trends were visualized graphically.

## 3 Results

### 3.1 Clinical characteristics and treatment timeline

The clinical characteristics and treatment sequences of our four patients, along with key reported cases from the literature, are comprehensively compared in **Table 1**. 100% of patients presented with typical KD manifestations. Three patients exhibited prominent joint symptoms, primarily limb swelling or pain. Patients 1–3 exhibited persistent febrile recurrences throughout the clinical course, while Patient 4 experienced a relapse of pyrexia complicated by polyarticular arthralgia and swelling after a 7-day afebrile interval. None had KD shock syndrome and MAS. All patients tested negative for COVID-19 nucleic acid or antigen. Patients 1 and 2 had coronary artery dilation before TCZ, including one with small aneurysms (Patient 1: Left coronary artery diameter: 0.3 cm, Z value: 2.08; Right coronary artery diameter: 0.3 cm, Z value: 2.49; Patient 2: Left coronary artery diameter: 0.38 cm, Z value: 4.6; Right coronary artery diameter: 0.28 cm, Z value: 2.9).

**Table 1:** Comparison of the present case series and reported cases in the literature.

Patient number	Sex	Age (years)	KOBAYASHI score	Treatment before the administration of TCZ					Therapeutic response	Coronary-artery dilatation before TCZ	Coronary-artery dilatation after TCZ	References
				1 Dose of IVIG	2 Dose of IVIG	Aspirin	Intravenous methylprednisolone	TCZ administration after onset(Days)				
1	M	3	7	Y	Y	Y	N	14	Recurrent fever	Y	N	Our case
2	M	2	2	Y	Y	Y	N	16	Recurrent fever	Y	N	Our case
3	M	3	7	Y	Y	Y	Y	17	Recurrent fever	N	N	Our case
4	F	4	2	Y	Y	Y	N	15	Relapse of pyrexia complicated by polyarticular arthralgia and swelling after a 7-day afebrile interval	N	N	Our case
5	F	1	ND	Y	N	Y	ND	7	Persistent fever	N	Y	21
6	M	8	ND	Y	N	Y	ND	7	Persistent fever	N	Y	21
7	M	4	ND	Y	N	Y	ND	8	Persistent fever	N	N	21
8	F	1	ND	Y	N	Y	ND	7	Persistent fever	N	N	21
9	M	3	6	Y	Y	Y	Y	8	Uncontrollable clinical symptoms	Y	Y	22
10	F	3	2	Y	N	Y	Y	10	Uncontrollable clinical symptoms Thrombocytopenia Hyperferriinemia	Y	N	22
11	F	3	6	Y	N	Y	Y	7	Uncontrollable clinical symptoms	Y	N	22
12	M	7	5	Y	N	Y	Y	7	Recurrent fever; Hyperferriinemia	N	N	22
13	M	2	2	Y	Y	Y	Y	9	Uncontrollable inflammatory storms	Y	N	22

The table summarizes key characteristics, including age, sex, timing of TCZ administration (days from disease onset), prior treatments, coronary artery status, and clinical outcomes.

M, Male; F, Female; Y, Yes;N, NO; ND, not described;IVIG, intravenous immunoglobulin; TCZ, Tocilizumab.

Initial IVIG treatment(2g/kg) and aspirin(30mg/kg) failed to control clinical symptoms. All patients received a second dose of IVIG prior to TCZ administration, but symptoms did not improve. In Patient 3, a second dose of IVIG was administered concomitantly with intravenous methylprednisolone at 5 mg/kg/day for 5 days.

A pivotal finding is the timing of TCZ administration: in our cohort, TCZ was initiated at a median of 15.5 days (range, 14–17 days) from disease onset, notably later than in historical cases (median 7–8 days).

### 3.2 Inflammatory marker response

**Figure 1:**
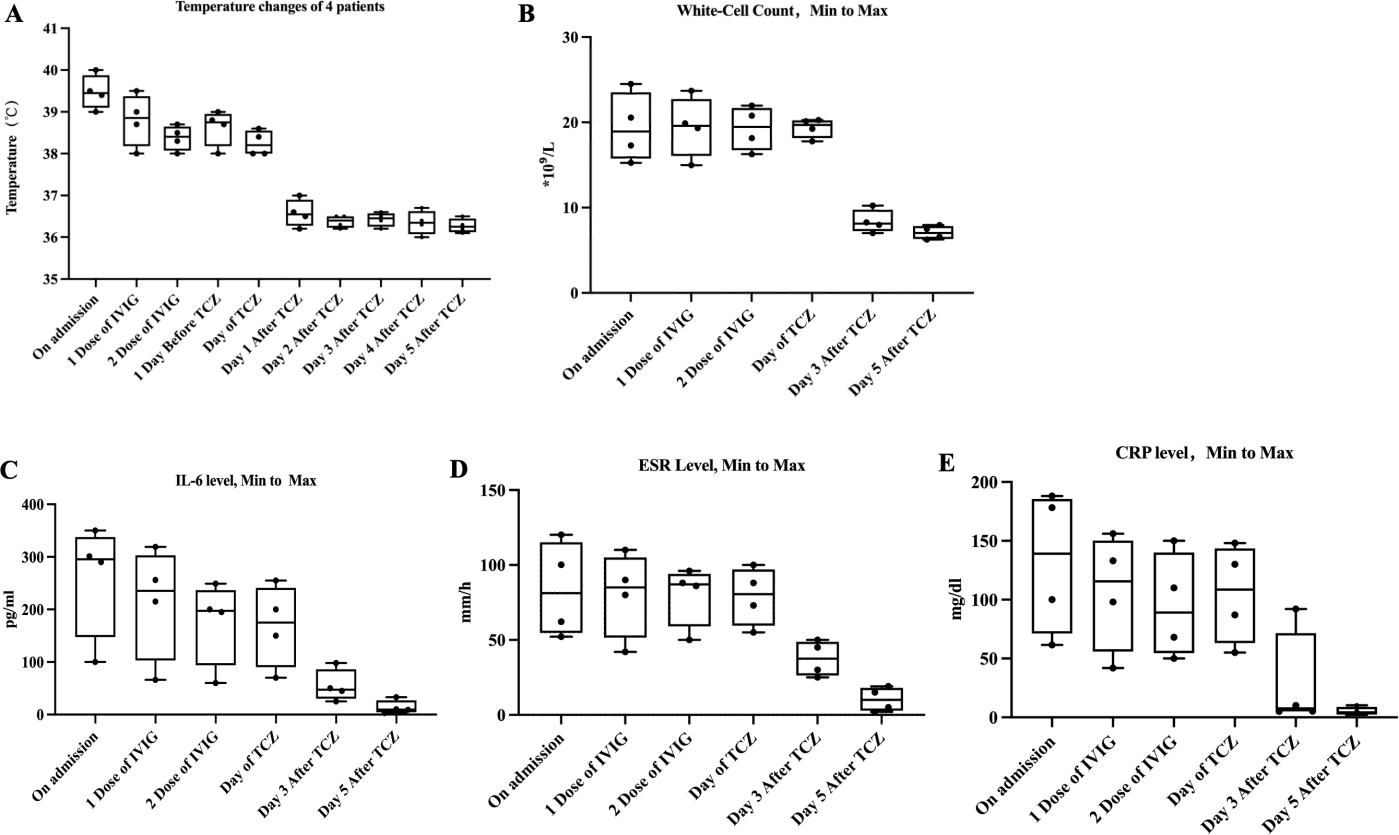
Clinical and laboratory response to TCZ in four patients with IVIG-resistant KD. **(A)** Body temperature curves for patients 1-4, showing daily maximum body temperatures from disease onset through the first five days after TCZ administration. All patients achieved normothermia within 24 hours of TCZ administration. **(B-E)** Serial measurements of key inflammatory markers: **(B)** C-reactive protein (CRP, mg/L), **(C)** Erythrocyte Sedimentation Rate (ESR, mm/h), **(D)** White Blood Cell count (WBC, ×10^9^/L), and **(E)** Interleukin-6 (IL-6, pg/mL). Following TCZ treatment, these inflammatory markers normalized quickly in all four patients.

Despite this later intervention, the clinical and laboratory response was rapid and uniform. All four patients became afebrile within 24 hours of TCZ infusion (**Figure 1A**). This was paralleled by a steep decline in acute-phase reactants, including White-Cell count、 interleukin-6 level、erythrocyte sedimentation rate(ESR)、and C-reactive protein(CRP), as detailed in **Figures 1B–E**.

### 3.3 Coronary artery outcomes

**Figure 2:**
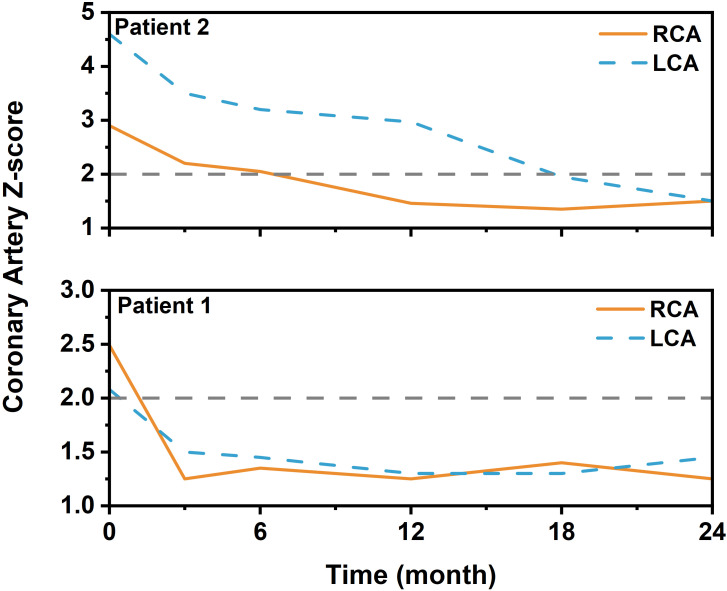
Longitudinal changes in coronary artery Z-scores in two patients (patients 1 and 2) with pre-existing lesions after late tocilizumab administration. Serial echocardiographic assessments show the dynamic changes in the maximum coronary artery Z-scores for patient 1 (complete resolution by 3 months) and patient 2 (progressive regression to normal by 18 months). The horizontal dashed line at Z = 2.0 indicates the upper limit of normal. LCA: left coronary artery; RCA: right coronary artery.

Coronary outcomes were favorable. Two patients (Patients 1 and 2 in **Table 1**) had pre-existing lesions. Patient 1 had coronary dilation (Left coronary artery (LCA) Z-scores 2.08; right coronary artery (RCA) Z-scores 2.49), which completely resolved to normal Z-scores (LCA Z-scores 1.5; RCA Z-scores 1.25) by the 3-month follow-up. Patient 2, presenting with a small aneurysm (LCA Z-score 4.6; RCA Z-scores 2.9), achieved complete normalization by 18 months (LCA Z-score 1.95; RCA Z-scores 1.35). Critically, no new coronary artery lesions developed in any patient during follow-up. The detailed trajectory of coronary Z-scores is provided in **Figure 2**.

### 3.4 Review of the literature and comparative analysis with present cases

To contextualize our findings, we conducted a review of published case series on the use of TCZ in IVIG-resistant KD. Our search identified two key studies providing patient-level data on coronary outcomes, encompassing 9 patients (Nozawa et al., 4; Ling et al., 5) (21, 22). **Table 1** provides a side-by-side comparison of all reported cases, including our 4 patients.

The case series by Nozawa et al. first raised a cautionary note regarding TCZ and coronary outcomes (21). In their report, two of four patients (Patients 5 and 6 in **Table 1**) developed new coronary artery aneurysms following TCZ therapy. Notably, these patients received TCZ relatively early (on day 7 of illness) after only a single dose of IVIG.

More recently, Ling et al. reported a five-patient series with mixed coronary outcomes (22). One patient (Patient 9 in **Table 1**), a 3-year-old boy with a high Kobayashi score of 6 and pre-existing CAL, experienced progressive coronary dilation after TCZ. This patient had received both a second IVIG and intravenous methylprednisolone (IVMP) before TCZ, indicating severe, multidrug resistance. The other four patients in their series, including three with pre-existing CALs, did not develop new lesions.

## 4 Discussion

The accurate diagnosis of IVIG-resistant KD is paramount yet challenging, as it shares clinical features with other systemic inflammatory conditions, notably sJIA and MAS. In this study, we employed a stringent diagnostic protocol (as detailed in the Methods) that incorporated the EULAR/PReS (2024) and EULAR/ACR (2022) criteria to exclude sJIA and MAS/HLH (19, 20), respectively, and that included comprehensive infectious workups. While this protocol significantly increases diagnostic confidence for KD in our cohort, we acknowledge an intrinsic limitation of retrospective studies: the potential for diagnostic uncertainty, particularly in the early, overlapping phases of these syndromes. The favorable response to IL-6 blockade observed here must therefore be interpreted within the context of patients who met rigorous KD criteria after exhaustive differential diagnosis.

During the acute phase of KD, proinflammatory cytokines, including TNF-α, IL-1, and IL-6, are markedly elevated. Studies have demonstrated that TNF-α antagonists and IL-1 receptor antagonists exhibit significant therapeutic effects in patients with IVIG-resistant KD (1, 23). Furthermore, emerging evidence from in-depth investigations into the pathogenesis of KD suggests that IL-6 overexpression plays a critical role in disease initiation and progression (24, 25). Given the pivotal role of IL-6 in the pathogenesis of KD, along with laboratory findings demonstrating markedly elevated serum IL-6 levels (>100pg/mL) in all patients, and considering the limited availability of IL-1 receptor antagonist therapies in China, our team ultimately elected to employ the IL-6 receptor antagonist (TCZ) as rescue therapy for these IVIG-resistant cases, which achieved rapid control of systemic inflammation. Notably, and in contrast to prior reports, none of our patients developed new CALs, and pre-existing CALs regressed. These compelling clinical observations can be mechanistically traced to the specific blockade of the IL-6 pathway by TCZ. The rapid resolution of fever and acute-phase reactants likely reflects the direct interruption of IL-6-mediated systemic inflammatory signaling. More importantly, the favorable coronary outcomes suggest that TCZ may not only suppress vascular inflammation but also uniquely promote a healing milieu, a hypothesis we explore below by examining its distinct mechanisms of action.

The pathogenicity of IL-6 in KD vasculitis mainly operates through its “trans-signaling” pathway. In this process, IL-6 binds to its sIL-6R, forming a complex that can activate vascular endothelial cells, which express the signal-transducing subunit gp130 but lack the membrane-bound IL-6R. This IL-6/sIL-6R complex on endothelial cells triggers a proinflammatory cascade, leading to the upregulation of adhesion molecules (e.g., VCAM-1, ICAM-1), increased recruitment of neutrophils and monocytes, and greater vascular permeability—ultimately resulting in endothelial dysfunction and the characteristic vasculitis of KD (26). TCZ, by competitively blocking the IL-6 receptor, directly inhibits this pathogenic trans-signaling cascade. We postulate that this direct endothelial protection is a key reason why no new coronary lesions emerged in our patients. The elevated IL-6 levels observed in our patients indicate a strong activation of this harmful pathway, making its blockade a logical therapeutic target.

**Figure 3:**
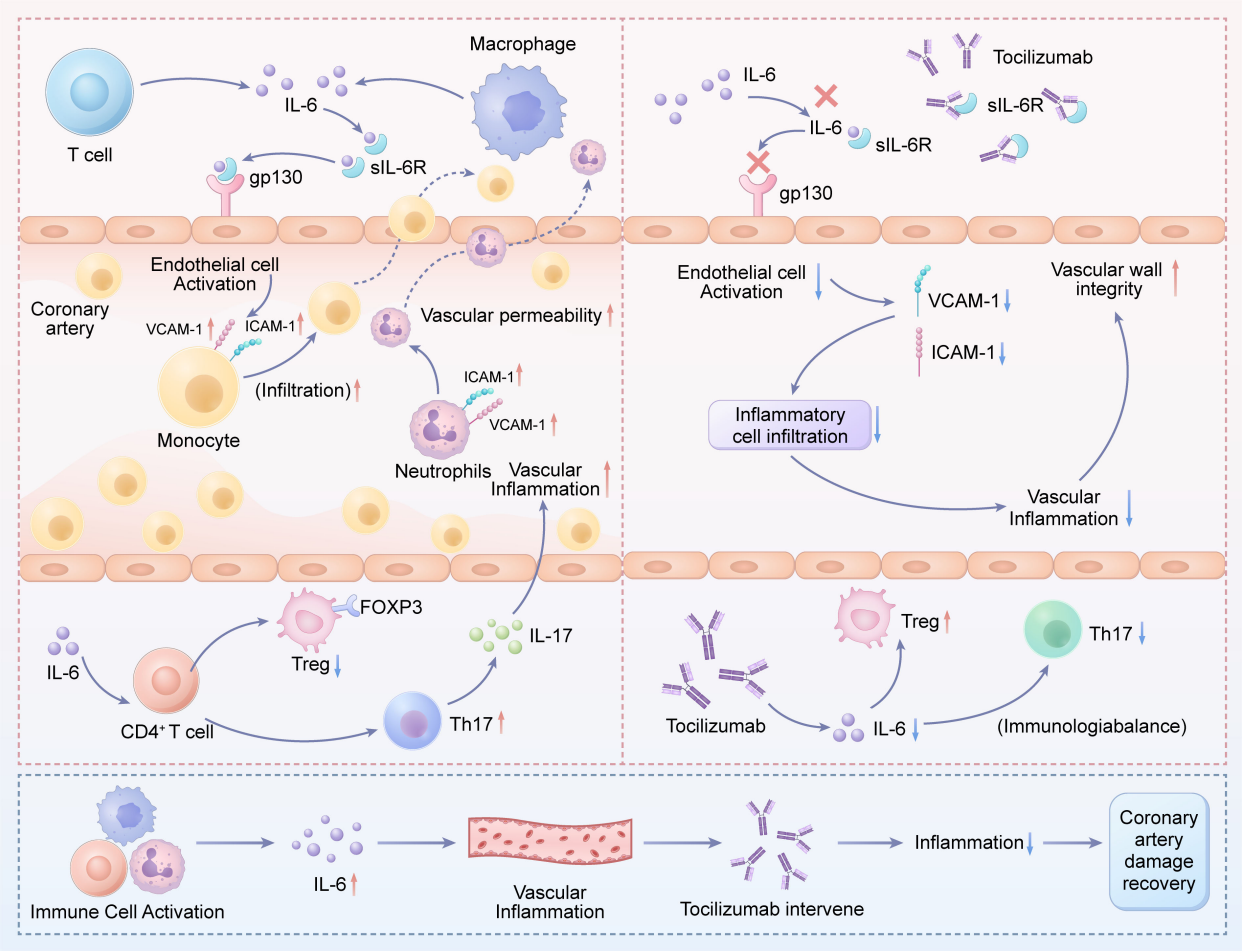
Proposed mechanism of action for TCZ in IVIG-resistant KD: Dual regulation of vascular inflammation and immune homeostasis. The schematic illustrates how excessive IL-6 production leads to coronary artery lesions via the trans-signaling pathway and disrupts the Treg/Th17 balance (left panel). TCZ, by blocking the soluble IL-6 receptor, prevents these harmful processes, thereby reducing vascular inflammation and potentially aiding coronary repair (right panel). IL-6, interleukin-6; sIL-6R, soluble IL-6 receptor; Treg, regulatory T cell; Th17, T helper 17 cell; gp130, glycoprotein 130; VCAM-1, Vascular Cell Adhesion Molecule-1; ICAM-1, Intercellular Adhesion Molecule-1; TCZ, tocilizumab.

Beyond direct endothelial activation, IL-6 serves as a key regulator of the adaptive immune response. Emerging evidence suggests that dysregulated immune activation in KD is closely linked to the suppression of Tregs during this phase. Specifically, IL-6 appears to downregulate FOXP3+ Tregs, leading to the polarization of proinflammatory Treg subsets and impairing Treg stability. This crucial reduction in Treg-mediated immunosuppression creates a permissive environment for the expansion of proinflammatory Th17 cells and other effector lymphocytes, which then release a surge of cytokines (e.g., IL-17) that directly target the vascular endothelium and exacerbate vasculitis (17, 18). This restoration of immune homeostasis by TCZ is critical for interpreting the observed regression of pre-existing CALs. By mitigating the IL-6-mediated suppression of Tregs, tocilizumab may have facilitated a shift from a proinflammatory to a reparative immune environment within the vascular wall, thereby enabling the structural remodeling and resolution of aneurysms observed during follow-up. This positions IL-6 blockade as having the potential for dual therapeutic effects: immediate anti-inflammatory and long-term pro-healing effects. The central role of IL-6 in the pathogenesis of IVIG-resistant KD and the mechanism of action of TCZ are summarized in **Figure 3**.

The therapeutic profile of TCZ in IVIG-resistant KD remains incompletely defined, with literature reports presenting a paradox regarding coronary outcomes. As summarized in **Table 1**, prior case series have documented instances in which TCZ was followed by the development or progression of coronary artery aneurysms (21, 22). Our observation of coronary aneurysm regression without new-onset lesions stands in contrast to these reports. Furthermore, preclinical studies in murine models of KD vasculitis suggest that while IL-6 is markedly upregulated, genetic or pharmacologic blockade of the IL-6 pathway does not prevent the development of coronary arteritis, indicating it may be more of a correlative “bystander” than a requisite driver in the initial disease phase (27). These apparently contradictory data across both clinical and preclinical domains challenge a simplistic model of IL-6 as a pathogenic target in KD. Rather than dismissing any set of findings, we propose that a critical, underappreciated variable may reconcile them: the disease stage at the time of intervention.

In our cohort, TCZ was administered significantly later (median 15.5 days from disease onset) than in the cases reported by Nozawa et al. (median day 7) and Ling et al. (median day 8). We hypothesize that the immunopathological landscape of KD evolves dynamically. The early hyper-acute phase (e.g., first week) may be dominated by a robust innate immune response and neutrophil-mediated vascular injury, a process in which IL-6, though elevated, may not be singularly critical—consistent with data from murine models (27). In this context, early IL-6 blockade might not fully abrogate vascular inflammation, potentially explaining reports of transient or progressive dilation. Conversely, the patients in our series represent a subacute, persistent inflammatory phase. By this stage, the pathophysiology may have shifted toward sustained adaptive immune dysregulation, in which IL-6 plays a pivotal role in maintaining a proinflammatory milieu (e.g., via Treg/Th17 imbalance) and, crucially, in suppressing vascular repair mechanisms. Therefore, we postulate that the efficacy and coronary safety profile of TCZ may be “phase-dependent”. Blocking IL-6 in this latter, dysregulated phase might be uniquely capable not only of suppressing inflammation but also of removing a barrier to intrinsic vascular healing, thereby facilitating the coronary remodeling we observed.

## 5 Conclusion

This case series demonstrates that late administration of TCZ (median 15.5 days from disease onset) in IVIG-resistant KD is associated with rapid control of systemic inflammation and, notably, favorable coronary remodeling, including aneurysm regression. These findings stand in contrast to prior reports of coronary dilation following earlier TCZ intervention.

To reconcile these disparate clinical outcomes, we integrate our data with the existing literature to propose a novel “phase-dependent” hypothesis. We postulate that the role of IL-6—and thus the therapeutic window for its blockade—evolves during the course of KD. In the early hyper-acute phase, IL-6 may act more as a bystander in neutrophil-dominant vasculitis. In contrast, in the persistent, subacute phase, it may become a key sustainer of adaptive immune dysregulation and impaired vascular repair. Blocking IL-6 in this later phase might therefore uniquely facilitate coronary healing.

This hypothesis reframes TCZ from a general anti-inflammatory biologic to a potential phase-targeted therapy. It provides a testable framework that explains conflicting evidence and directs future research. The key clinical insight is that the timing of intervention may be a critical determinant of success. While prospective validation is needed, this concept offers a practical guide for therapeutic decision-making and a clear rationale for designing future trials stratified by disease stage in refractory KD.

## 6 Future research directions

Several limitations of this study must be acknowledged, primarily stemming from its retrospective design, small sample size, and lack of a comparative control group, which constrain the generalizability of our findings and the strength of causal inference. To definitively establish the role of IL-6 blockade in IVIG-resistant KD, future research should transition from observational reports to hypothesis-driven investigations. We propose the following specific directions:(1) Definitive Efficacy and Safety Trials: The highest priority is a multicenter, randomized controlled trial (RCT) comparing TCZ directly against current standard rescue therapies (e.g., a second IVIG infusion, intravenous corticosteroids, or infliximab). (2) Optimization of Clinical Protocols: Our study, alongside others, highlights unresolved practical questions. Prospective studies are needed to determine the optimal therapeutic window for TCZ administration—whether earlier intervention in all refractory cases is superior, or if a later intervention in persistently inflammatory cases is more beneficial for vascular healing. (3) Pharmacokinetic/pharmacodynamic studies: pharmacokinetic/pharmacodynamic studies in children with KD are essential to define the optimal dose and interval of TCZ, moving beyond protocols borrowed from other diseases like sJIA; (4) Long-term coronary artery Follow-up: The regression of aneurysms in our series is promising but requires long-term confirmation. (5) Furthermore, alongside clinical efficacy, future studies must also consider the pharmacoeconomic impact and healthcare resource utilization of different treatment strategies for refractory KD, as highlighted in recent multinational analyses (28).

## Data Availability

The original contributions presented in the study are included in the article/supplementary material. Further inquiries can be directed to the corresponding authors.
